# Increased population size of fish in a lowland river following restoration of structural habitat

**DOI:** 10.1002/eap.1882

**Published:** 2019-04-04

**Authors:** Jarod P. Lyon, Tomas J. Bird, Joanne Kearns, Simon Nicol, Zeb Tonkin, Charles R. Todd, Justin O'Mahony, Graeme Hackett, Scott Raymond, Jason Lieschke, Adrian Kitchingman, Corey J. A. Bradshaw

**Affiliations:** ^1^ Department of Environment, Land, Water and Planning Arthur Rylah Institute for Environmental Research 123 Brown Street Heidelberg Victoria 3084 Australia; ^2^ School of Biological Sciences University of Adelaide Adelaide South Australia 5005 Australia; ^3^ School of Botany University of Melbourne, School of Botany Melbourne Victoria 3010 Australia; ^4^ Institute for Applied Ecology University of Canberra Bruce Australian Capital Territory 2617 Australia; ^5^ Australian Bureau of Agricultural and Resource Economics and Sciences Department of Agriculture and Water Resources GPO Box 858 Canberra Australian Capital Territory 2601 Australia; ^6^ Global Ecology College of Science and Engineering Flinders University GPO Box 2100 Adelaide South Australia 5001 Australia

**Keywords:** citizen science, coarse woody habitat, golden perch, meta‐population, Murray cod, resnagging, scale, stream restoration

## Abstract

Most assessments of the effectiveness of river restoration are done at small spatial scales (<10 km) over short time frames (less than three years), potentially failing to capture large‐scale mechanisms such as completion of life‐history processes, changes to system productivity, or time lags of ecosystem responses. To test the hypothesis that populations of two species of large‐bodied, piscivorous, native fishes would increase in response to large‐scale structural habitat restoration (reintroduction of 4,450 pieces of coarse woody habitat into a 110‐km reach of the Murray River, southeastern Australia), we collected annual catch, effort, length, and tagging data over seven years for Murray cod (*Maccullochella peelii*) and golden perch (*Macquaria ambigua*) in a restored “intervention” reach and three neighboring “control” reaches. We supplemented mark–recapture data with telemetry and angler phone‐in data to assess the potentially confounding influences of movement among sampled populations, heterogeneous detection rates, and population vital rates. We applied a Bayesian hierarchical model to estimate changes in population parameters including immigration, emigration, and mortality rates. For Murray cod, we observed a threefold increase in abundance in the population within the intervention reach, while populations declined or fluctuated within the control reaches. Golden perch densities also increased twofold in the intervention reach. Our results indicate that restoring habitat heterogeneity by adding coarse woody habitats can increase the abundance of fish at a population scale in a large, lowland river. Successful restoration of poor‐quality “sink” habitats for target species relies on connectivity with high‐quality “source” habitats. We recommend that the analysis of restoration success across appropriately large spatial and temporal scales can help identify mechanisms and success rates of other restoration strategies such as restoring fish passage or delivering water for environmental outcomes.

## Introduction

Fish populations are imperiled globally (Dudgeon et al. 2006) and under increasing threat from human population growth and climate change (Pauly et al. 2002, Ficke et al. 2008). As such, fishery and conservation managers work closely with fishery biologists to implement programs that aim to restore rivers and protect fish populations from further decline (Trexler 1995, Palmer et al. 2005). To aid these restoration efforts, an understanding of the underlying processes that limit populations of fish within modified riverine environments is required (Schlosser 1991, Fausch et al. 2002). Fish populations change in time due to variability in processes such as connection to a catchment for input of organic material (e.g., the river continuum concept; Vannote et al. 1980), and the availability and heterogeneity of habitats and their connections within a patchy “source” and “sink” framework (e.g., the dynamic landscape model for stream fish ecology developed by Schlosser 1991). However, quantifying the relative influence for fish populations of altering or restoring different processes and habitats, in a way that is useful for managers who need to prioritize restoration budgets with some degree of certainty about likelihood of success, remains difficult (Fausch et al. 2002, Allan 2004, Bernhardt et al. 2005).

A well‐identified problem is that demonstrating tangible outcomes of restoration, particularly at broad spatial scales (more than hundreds of kilometers) has been challenging (Bernhardt et al. 2005, Lepori et al. 2005, Palmer et al. 2005). Indeed, most rigorous assessments of ecological outcomes have focused on small restoration projects where covariates are easier to control/measure (Lake 2001, Palmer et al. 2005, Sass et al. 2017). The typically long time frames of many ecological responses seldom align with the shorter windows of funding programs (Bernhardt et al. 2005, Lindenmayer et al. 2012, Cooke et al. 2017), and it can be difficult to disentangle the effects of management interventions from those arising from other environmental drivers (e.g., climate variation) without appropriate spatial replication, reference systems, pre‐intervention monitoring and/or extensive time series data (Likens 1989, Carpenter 1998, Arthington et al. 2006, Palmer et al. 2010).

One strategy increasingly applied is to shift effort from monitoring many smaller interventions to assessing fewer, longer‐term restoration projects and investing more resources into well‐designed monitoring programs (Callahan 1984, Lindenmayer et al. 2012, Lohner and Dixon 2013). This approach assumes that learning from a few well‐monitored interventions will provide more robust evidence for effective application of the interventions elsewhere (Swirepik et al. 2015). However, apart from notable programs such as the Upper Mississippi (Braun et al. 2016) and Rhine (Verweij 2017) rivers, examples of broad‐scale river restoration successes backed by rigorously designed monitoring, at sufficiently large enough spatial and temporal scales, remain scarce (Fausch et al. 2002).

Fish are useful indicators of ecological processes, such as carbon uptake occurring at lower trophic levels (Tonkin et al. 2017), and are highly valued by society for subsistence, commercial, and recreational purposes (Feather et al. 1999). Furthermore, fish often need ecological restoration following anthropogenic disturbances to waterways that exclude them from optimal habitats (Collares‐Pereira and Cowx 2004, Dudgeon et al. 2006). Globally, restoration and threat abatement to halt and reverse these declines include restoring fish passages, removing pests, providing water to achieve environmental outcomes, restocking, revising fishing regulations, changing land practices, and restoring habitats (Dudgeon et al. 2006). One restoration method is to reintroduce coarse woody habitat to areas where it has been historically removed. Coarse woody habitat plays many roles within stream networks (Zalewski et al. 2003), and in lakes and reservoirs (Sass et al. 2012). For riverine fishes, coarse woody habitat provides habitat and protection for feeding, shelter and spawning (Schlosser 1991, Crook and Robertson 1999, Tonkin et al. 2016). Given the ecological importance of coarse woody habitats, it is logical to test the response of target fish populations to their restoration. While Schlosser (1991) provided a framework where spatially separated heterogeneous habitats are important to support different fish life history stages within a “patchy” lotic environment, testing in large, lotic systems is difficult, but increasingly necessary, to justify restoration investment.

At broad spatial scales, predatory fish in larger lowland rivers comprise connected populations that interact and influence one another (Levins 1969, Schlosser 1991, Albert and Reis 2011). Variable movement and recruitment, variable sampling efficiency and environmental conditions among them often lead to extreme differences in abundance estimates that might mask the true response of fish species to localized restorations. Accounting for these population differences and their connectivity is therefore required to determine restoration success. We thus applied a conceptual model (Albert and Reis 2011) that links the fish in a reach of the Murray River in south‐eastern Australia where coarse woody habitat was restored resnagging, with three neighboring populations in control reaches. Using the *a priori* predictions of the conceptual model (that our intervention would increase the number native fish in the total population), we designed and implemented a large research and monitoring program and used the data outputs from this program to model the changes in population size of two large, native, predatory fish species — Murray cod (*Maccullochella peelii*) and golden perch (*Macquaria ambigua*) — over seven years following restoration of a historically desnagged reach with coarse woody habitat. Our aim was to test the hypothesis that restoring coarse woody habitats at a reach scale (>100 km) in a large, lowland river results in a net increase in population size for two target species of native fishes, rather than merely redistributing fishes already present.

## Methods

### Ecosystem setting

The Murray River is Australia's longest river and forms a major component of the Murray‐Darling Basin that has a catchment area of 1.07 million km^2^ (Walker 1992). Flow is highly regulated for irrigation, with large impoundments on the Murray River and its tributaries (Walker 1992). The natural flow regime used to be highly variable and was characterized by peak flows in winter and spring, with low flows in summer and autumn. Flow regulation has since reversed the seasonal pattern such that flows peak in summer and are lowest in winter, although some variability remains (minimum flow is 25% of mean, maximum is over 200% of mean; Rutherfurd 1991).

### Study species and populations

The two species in this study are medium‐ to large‐bodied native freshwater fish (Murray cod [>50 kg and 1,400 mm maximum size, with females reproductively mature at 500 mm length with up to 100,000 eggs produced in large females; see Appendix S1: Plate S1], and golden perch [>10 kg and 600 mm maximum size, with females reproductively mature at 300 mm length with up to 500,000 eggs produced in large females]) with a strong association with instream habitats that they use for refuge, cover for ambushing prey, and spawning sites (Koehn and Nicol 2014). These species are also long‐lived; Murray cod can live up to 48 yr (Anderson et al. 1992), and golden perch up to 26 yr (Mallen‐Cooper and Stuart 2003). Both species have been reduced in abundance and range since European settlement (Lintermans 2007).

**Figure 1:**
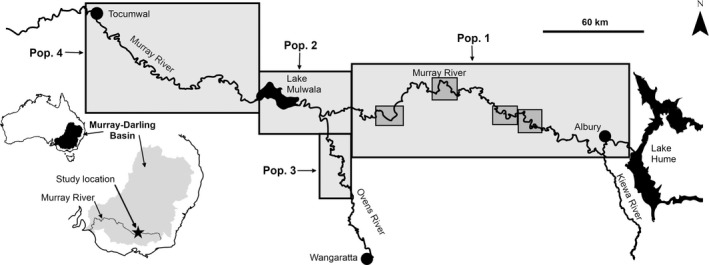
The study location in south‐eastern Australia. Pop. = population. Priority resnagging zones are indicated by gray shaded boxes within population 1. Yearly number of sites sampled in each population can be found in Appendix S1: Table S2.

Our study populations can be described as an “intervention” population in a river reach where structural woody habitat restoration was done and three “control” populations in river reaches without structural restoration. These populations had varying connectivity (Fig. 1). The “intervention reach” is in a section of the Murray River approximately 120 km from Lake Hume to the junction of Lake Mulwala. The intervention was done between the years 2007 and 2010 and involved reintroducing 4,450 large (mostly >1 Mg) pieces of coarse woody habitat sourced from natural trees recovered from a large road project, within four, 5,000‐m priority zones between 25 and 100 river km downstream from Lake Hume (Fig. 1; Appendix S1: Plates S2, S3). Data collection in this reach began in 2007, prior to structural woody habitat restoration, and forms the basis for a “before” baseline data set for an unreplicated before/after‐control/impact (BACI) design.

The three “control” populations were as follows. Population 2 is in control reach 1, immediately below the intervention reach in Lake Mulwala and its tailwaters into the Murray and Ovens Rivers. This reach is formed by a 7‐m weir used to divert water for irrigation and covers an area of 4,450 ha when full. The Ovens River flows into the intervention reach approximately 5 km above its confluence with Lake Mulwala (Fig. 1). Population 3 is in control reach 2 and occupies the 80‐km reach of the Ovens River between the township of Wangaratta and the rivers confluence with Lake Mulwala. Population 4 occupies control reach 3, a 100‐km reach of the Murray River immediately downstream of Lake Mulwala extending to the township of Tocumwal.

### Habitat description

Fish passage between populations 1, 2, and 3 is unrestricted in all directions (Koehn et al. 2009). Thus, populations 2 and 3 are potential source populations for changes to population 1 in response to coarse woody habitat restoration. While fish can pass downstream to population 4 over weir gates or upstream from population 4 via a fish lift, movement is restricted compared to background rates (Stuart et al. 2010).

Habitat types in the intervention reach (population 1) range from shallow, fast‐flowing sections nearer Lake Hume to slow‐flowing, deeper pools (>4 m) closer to Lake Mulwala (Fig. 1). This reach is degraded, with poor riparian and instream habitats and altered flow regimes. Over 25,000 large pieces of coarse woody habitat were removed from this reach from 1950 to 1980 to improve water conveyance. In control reaches 1 and 2 (populations 2 and 3), depth (maximum depth = 14 m), flow, and turbidity characteristics differ from the other study reaches, and hydrology in the lower section of control reach 2 (population 3) is influenced by water levels in Lake Mulwala. A lake drawdown affected this hydrology during the sampling period of 2011 and might have impacted abundance estimates for populations 2 and 3 for that year by changing capture efficiency. Some removal of coarse woody habitat occurred historically in control reach 3 (population 4); however, the instream habitat is comparatively undisturbed, with naturally occurring woodfall in the reach (Nicol et al. 2004). The depth characteristics of control reach three resemble those in the intervention reach. When irrigation offtakes exceed Ovens River inflow, the presence of Lake Mulwala leads to lower flow volumes in control reach three than in the intervention reach; however, the shape of the hydrograph remains essentially the same (Appendix S1: Fig. S1).

The short distances between each of the populations (<50 km) mean that they experienced similar climatic variation over the study period. Despite the Ovens River (control reach 2, population 3) being unregulated, river discharges and temperatures follow comparable trajectories across all reaches over the study period (Appendix S1: Fig. S1). River discharges and water temperatures had high inter‐annual variation, primarily driven by reduced inflows from 2007 to 2010 associated with the “Millennium drought,” severe flooding in 2010–2011, and the return of long‐term average conditions during the final two years of the study (Dijk et al. 2013).

Given this spatial arrangement, we assumed background fluctuations in target species abundance (outside those being measured in response to our resnagging intervention), to be consistent across the treatment and control reaches. Given tagging occurred on larger fish (which generally have lower natural mortality), we assumed that survival estimates among populations would be similar (Pauly 1980).

### Monitoring design

We designed our monitoring approach to estimate annual changes over seven years in population size of two native fish species within the four study populations (Appendix S1: Table S1) using data collected from a temporally and spatially large field study based on measurements to inform the population density function of *N*_*t*+1_ = *N*_*t*_ + (births − deaths) + (immigration − emigration). We used a mark–recapture framework to estimate these population parameters and vital rates for each species. We used electrofishing as our primary sampling method, which we then augmented with other sampling measures to account for varying sampling efficiency and to estimate fish movement between reaches and mortality. To account for these potential sources of bias, we modified our sampling program by using (1) multiple sources of capture information, (2) focused experiments to characterize capture rates, and (3) radio‐tagging technology to estimate mortality and movement rates between reaches.

We used electrofishing surveys (Smith‐Root generator‐powered pulsator boat‐mounted electrofishing units using pulsed DC; Smith‐Root, Vancouver, WA, USA) during daylight hours within discrete sites randomly located within our study reaches. Within the intervention reach, sites were inside and outside the four priority resnagging zones; (Fig. 1) to account for the possibility that the fish already present in the intervention reach simply moved to and occupied the new habitat, leaving the original habitat unoccupied. Sites (150–250 m long, or about 1 ha) were sampled from 2007 to 2013 across the four populations (Appendix S1: Table S2). Surveys were done between April and June each year because reduced river discharge and flow variability at this time of year (i.e., end of the irrigation season; Appendix S1: Fig. S2) maximized electrofishing sampling efficiency (Lyon et al. 2014).

### Mark–recapture

We collected fish with a single pass of the electrofishing unit that sampled all available habitats (described in more detail in Nicol et al. 2004, Nicol 2005). We weighed each captured fish to the nearest gram and measured its total length to the nearest millimeter. To minimize error from tag loss, we double‐tagged fish by inserting a uniquely coded external t‐bar or dart tag adjacent to the dorsal fin on the left side of each fish >200 mm in total length and inserted a passive integrated transponder (PIT) tag into the stomach cavity. The external tags clearly displayed details for anglers to report relevant capture data (species, date, place of capture, and fish length). Annual electrofishing efficiency studies informed our estimates of detectability across each species, sizes and environmental gradients (more detail provided in Lyon et al. 2014).

### Citizen science

While electrofishing provided a replicable and robust basis for our mark–recapture program, recapture rates were variable. Given potentially high rates of angling pressure in our study populations (J. Lyon, *unpublished data*), we sought help from recreational anglers to produce additional data to reduce uncertainty in parameter estimates. We used an angler phone‐in program to collect tag return, location, size, and mortality data from the public (via the details provided on our externally tagged fish). Combining tag‐reporting by citizen scientists with mark–recapture studies has previously improved parameter estimation (Barker 1997, Barker et al. 2004).

### Radio tagging

Mark–recapture models assume a “closed” population where all animals are available for capture within a survey site between one sampling time and the next (Pradel 1996). In addition, angler and electrofishing data can only be collected from live fish, meaning that mortality must be inferred when using standard techniques. Furthermore, mark–recapture estimates of populations can be biased by movement between reaches. To account for inter‐reach mobility and to estimate mortality more reliably, we implanted a subsample (1,159 individual fish from the main electrofishing sample; Appendix S1: Table S3) with radio transmitters (150 MHz frequency; Advanced Telemetry Systems, Isanti, Minnesota, USA; see methods outlined in Barrett 2004). The size of implanted radio‐transmitters depended on the weight of the fish, with the proportion of transmitter mass restricted to <1.5% of each fish's body mass in air to avoid compromising fish buoyancy. This ratio of transmitter mass to body mass has minimal effect on survival rates (Saddlier et al. 2008, Bird et al. 2017). Battery life depended on the size of the transmitter, ranging from 45 to 1,200 d. We coded 20% of the transmitters with an 11 month off/1 month on cycle to save battery life (and hence enable smaller transmitter size), so that mortality of smaller fish could be tracked for longer periods (i.e., these fish were tracked for mortality signals during the one‐month “on” period). We also marked radio‐tagged fish with external t‐bar/dart tags to account for mortality from removal by anglers.

To improve estimates of survival and movement rates derived from the mark–recapture study, we recorded radio‐tagged fish via an array of fixed radio towers to monitor movement between populations and did annual tracking surveys to estimate mortality rates (via the mortality switch in the transmitters). Annual censuses of radio tags revealed fish location and mortality across all four study reaches. Given that fish often moved between reaches during foraging or spawning movements, we condensed data to what we describe as lasting relocations to inform our model, i.e., we categorized change as having occurred when an individual moved into a new reach for the majority of a given sampling year.

### Bayesian state‐space model

Our main parameter of interest was the number of fish of each species present in the four different populations. Under conditions where capture probabilities are invariant between individuals and years, population size can be estimated via capture‐recapture methods. However, one of the basic assumptions of capture‐mark‐recapture analyses is that individuals in a population have homogenous probability of recapture. In the Murray River (and indeed in many such populations), fish can transition between population areas that have substantially differing sampling characteristics, as well as different ecological conditions. As well, individuals can grow throughout the study, resulting in variable recapture rates through different life‐stages (Lyon et al. 2014). Our survey design consisted of four independent data sets: two based on observations from passive tags alone and two based on radio telemetry. While various methods accommodate each of these desired analytic outcomes separately, no “off‐the‐shelf” capture‐mark‐recapture package exists to allow full representation of the data in our study.

To estimate population size, we implemented a state‐space Cormack‐Jolly‐Seber model (King 2012) in the JAGS programming language (Plummer 2003) using the R statistical package (R Development Core Team 2013), which accounted for the effects of individual variation in capture probabilities while supplementing standard capture‐recapture data with angler tag recoveries and telemetry data. In total, the model consisted of multiple concurrent “state” models for individual survival, location and age, along with four “process” models to model the observed captures and individual lengths given the partially observed states. First, we modelled the yearly location of individuals within each of the 4 reaches as a categorical random variable, with one category per reach. Next, we estimated parameters of a von Bertalanffy growth curve to observed changes in length for recapture fish. From these growth curve parameters, we were able to back‐calculate estimated ages at first capture for all fish in the study (Bird et al. 2019). Estimated ages were then used as a covariate to help account for how individual capture and survival probabilities varied over time. Once we had accounted for these variables, we were then able to use the estimated capture rates to calculate what fraction of the true population had been sampled and therefore, the true size of the total population in each year, as per Bird et al. (2014). Full details of the model structure and data are available in the supplementary methods section (Appendix S1: Note S2).

## Results

### Mark–recapture

We captured 7,312 Murray cod and 3,743 golden perch between 2007 and 2013 during our electrofishing census. Of these fish, we tagged 3,839 Murray cod and 3,316 golden perch with PIT and external tags, and 689 Murray cod and 466 golden perch with radio tags (Appendix S1: Tables S3, S4). Over the study, anglers reported capturing 1,338 tagged Murray cod and 275 tagged golden perch from our study reaches. Multiple size classes were represented in the Murray cod population; however, there was a clear effect of angling on larger cohorts with a sharp decline in abundance of fish over 500 mm (>500 mm are subjected to angling pressure; Appendix S1: Fig. S2), while golden perch were represented primarily by larger cohorts (>300 mm; Appendix S1: Fig. S3).

**Figure 2:**
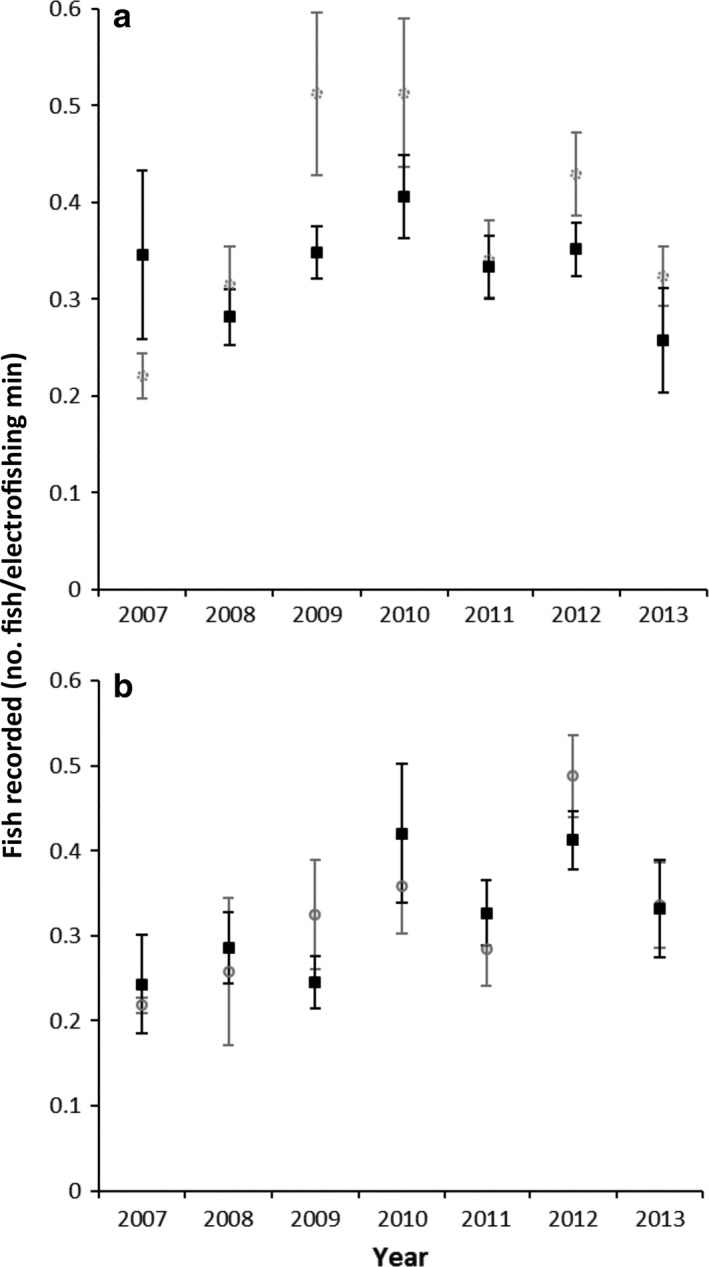
Number (mean ± SE) of (a) Murray cod and (b) golden perch recorded per minute during annual electrofishing surveys within the population 1 for sites within the priority resnagged zones (filled boxes) and sites outside the priority resnagged zones (shaded boxes) from 2007–2013. Electrofishing surveys were done following methods outlined in Nicol et al. (2004).

Within our intervention reach, we observed no large shifts in electrofishing catch per unit effort among sites within resnagged priority zones and sites that were in areas outside the priority resnagged zones (Fig. 2).

### Population demographics

**Figure 3:**
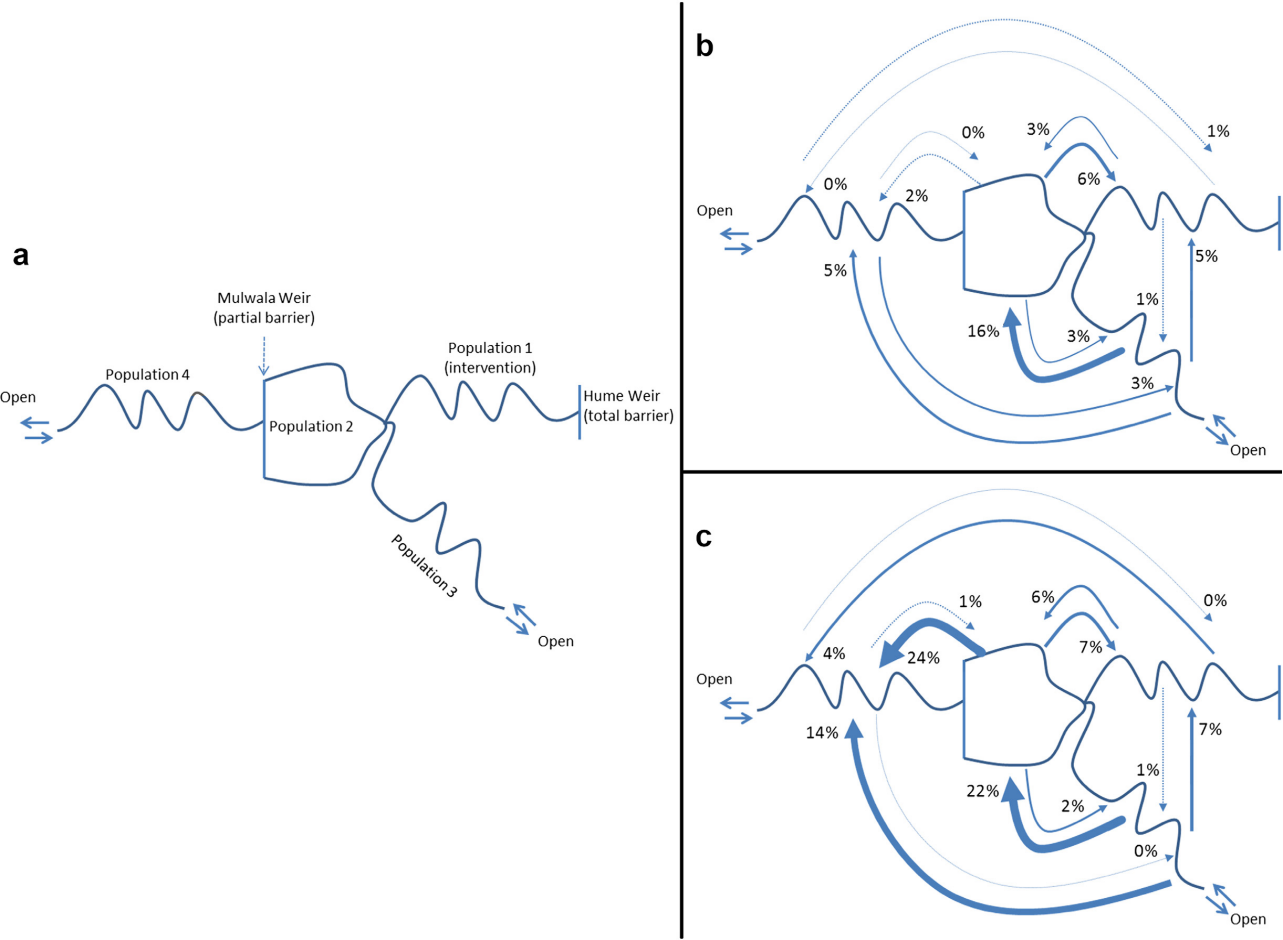
(a) Conceptualization of the population and its transition probabilities (permanent movement within the study period measured in percentage of total radio‐tagged population) for (b) Murray cod and (c) golden perch. Thickness of line is approximately proportional to scale of permanent transition.

Both target species were mobile, and there was exchange of individuals among study reaches. More than six million records were registered on our logging stations, which we condensed into 558 permanent location changes (i.e., most fish temporarily emigrated then returned to their population of origin). Of our subsample of radio‐tagged animals, 36 more fish immigrated into than emigrated out of the intervention reach (population 1). Conversely, there was a net decrease in immigration to populations 2 and 3 combined (49 fish), while there was a net increase in immigration to population 4 (13 fish). Fish moved among all three reaches, with most moving upstream from populations 2 and 3 into population 1. Another seven individuals migrated upstream out of population 4 using the fish lift, and into population 1. When scaled up to the population level (% of each population transitioning over the study period), populations 2 and 3 proved to be net sources of fish, contributing many adult recruits to surrounding populations (Fig. 3). Mulwala Weir formed an almost total barrier to upstream movement, but downstream movement was higher, with many golden perch moving during the 2011 flood (Fig. 3).

For both species, the model predicted that survival within population 1 remained approximately constant across years (Appendix S1: Fig. S4). In population 4, survival probabilities were generally stable, increasing slightly over time. Populations 2 and 3 had variable survival probabilities and associated errors due to fewer tagged fish and fewer recaptures.

### Population trends

**Figure 4:**
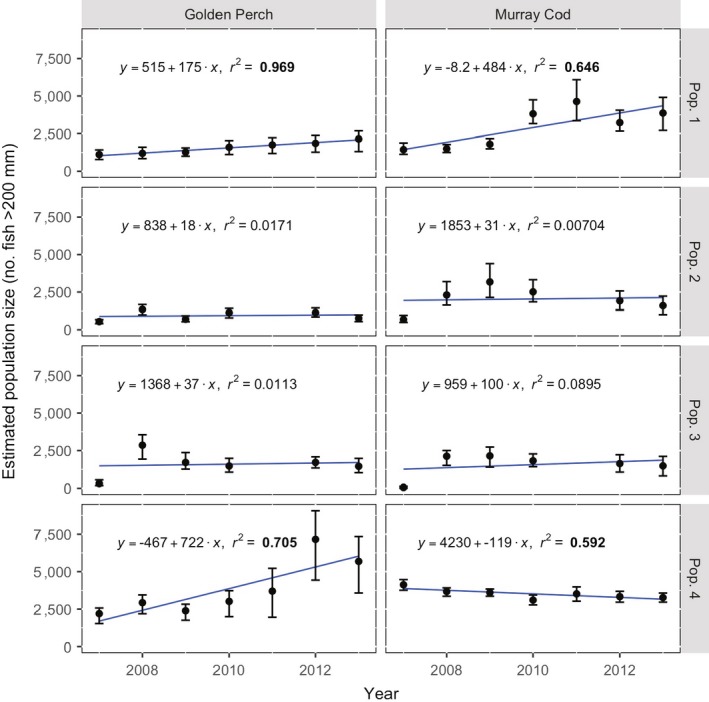
Estimated population size of Murray cod and golden perch (estimated total number of fish >200 mm in sampling sites). Error bars are 95% credible intervals, blue lines indicate linear trends over the course of the study, with non‐random correlations highlighted in boldface type (*P* < 0.05).

The model estimated that the population size of Murray cod (>200 mm within our sample sites) showed an increasing (*P* < 0.0001) trend after coarse woody habitat restoration (between 2007 and 2013) in population 1 (the intervention reach), with a 40% decrease (*P* < 0.05) over the same period in population 4 (which experienced the most similar geomorphic and hydrologic features, but was isolated from population 1 by a weir; Fig. 4). There was no evidence for a trend in abundance for populations 2 and 3 over the same period, with a spike observed in 2011 coinciding with the lake drawdown (and hence, potentially increased detection). Regardless, the peak in population 1 does not coincide with a decline in either populations 2 or 3, suggesting growth in the Murray cod population size. Between 2007 and 2011, our sites in the intervention reach (population 1) held around 1,000 golden perch >200 mm and increased (*P* < 0.05) up to around 2,000 animals over the course of the study. Golden perch abundance in population 4 was stable from 2007 to 2010, then roughly tripled following the 2010–2011 floods, mainly through adult fish immigrating during the flood year (see yearly catch *n* in Appendix S1: Fig. S3).

## Discussion

We found that population size of target species in our “intervention” (population 1) reach increased following habitat restoration in a large, lowland river. Movement of Murray cod and golden perch between reaches led to increased occupancy of the river reach where habitat was restored. The connectivity between our study reaches was (Tables S4 and S5, Fig. 3) an important predictor, with most immigrants arriving from the closest adjacent populations where no barriers to movement were present. Increases observed in the intervention reach were unlikely to be due to fish moving from within the intervention reach into the resnagging priority zones or, if this did occur, the previously vacated habitats were then occupied by different fish (Fig. 2).

### Role of connectivity and scale

At large spatial scales, fish populations can act as metapopulations, where connected populations interact and influence one another (Levins 1969). In our study, transfers from source populations (those in control reaches 1 and 2) into the restored reach exceeded other movement rates and enabled an increase in numbers of adult fish of both species in the intervention reach after habitat restoration. Palmer et al. (1997) first posed the “field of dreams” hypothesis (“if you build it, they will come”) and the importance of connectivity to a source population to explain the success of restoration projects across multiple spatial and temporal scales (Jansson et al. 2007). Hanski (1998) also provided a framework to describe how suitable source populations can fulfil the “replacement condition”: that one population of animals occupying high‐quality habitat can provide an overflow of recruits to “top up” another population experiencing a recruitment or survival bottleneck. These two frameworks clearly applied to both species in our study. While it is unlikely that fish are moving in response to the restored habitat, we contend that during foraging or “ranging” behavior, fish might come across unoccupied areas of newly installed habitat (or newly vacated habitat if another fish has vacated an already present habitat to occupy a new one, creating an occupancy in the process) and then occupy it, adding to the population as described here (Schlosser 1991). Furthermore, in control reach 3, which is the most geomorphologically similar, but is largely disconnected from our “source” population in Lake Mulwala (Fig. 3), populations of Murray cod declined over the period of our study, likely in response to lack of stream productivity during the “Millennium Drought” observed in southeastern Australia during this time, but that broke in 2011 (Dijk et al. 2013). While golden perch in control reach 3 did increase in numbers over the study period, this was largely due to a large immigration event in 2010/2011 driven by floods prompting movement of adult golden perch to this reach and increasing the population (Fig. 4).

Quantitatively demonstrating the ecological processes that lead to successful restoration has become increasingly important for the restoration of degraded rivers around the world (Konrad et al. 2011, Davies and Gray 2015, Roberts et al. 2016, Turunen et al. 2016). Our results provide support for the dynamic‐landscape model of life history of stream fish described by Schlosser (1991). An important consideration for successful restoration is the capacity to match restoration outcomes to target organisms (Fausch et al. 2002, Bond and Lake 2005, Sundermann et al. 2011), and the spatial scale of restoration and the temporal scale of the monitoring need to match the life history of the target organism (Palmer et al. 2010). Here, the target species had a home range of >100 km, the investment and scale of the restoration was large, our objective was to increase the population size of native predatory species, and we had a monitoring program designed to measure such changes at the relevant scales.

### Habitat heterogeneity

Restoring environmental heterogeneity in disturbed riverscapes is one of the most important elements in maintaining and enhancing fish populations in riverine habitats (Schlosser 1991, Sass et al. 2017), because variability in habitats across reach scales means that large‐bodied predatory species can complete their life histories. Habitat heterogeneity also ensures that resident individuals can react to physical habitat changes by moving among populations (Schlosser and Angermeier 1995). In our case, the altered flow regime and history of habitat degradation within the intervention reach mean that there was unlikely to be sufficient heterogeneity to support a spawning stock enough to offset mortality. However, we found that the population inhabiting the intervention reach is part of a larger meta‐population that provides overflow recruits via immigration to counter this lack of localized recruitment, while still fulfilling its own replacement condition. This can lead to an increase in carrying capacity across the meta‐population (Lipcius et al. 2008).

## Conclusion

We have demonstrated that a large‐scale management intervention promoted a response from our target species by increasing the available structural habitat, effectively increasing the population size for target fish in the intervention river. However, success also relied on connectivity between population (i.e., Tables S4 and S5; Fig. 3) that enabled transition of recruits into the restored reach. Populations of the target fish species here have declined over the past 200 yr (Lintermans 2007), and considerable investment is now being made to restore them using a variety of management “levers”, such as provision of environmental water, fish restocking, and construction of fishways. However, given the size of the rivers in question, and the scale of the restoration required, a continuing quandary for fisheries and resource managers is that the systems most in need of restoration are most often the largest (e.g., Mississippi and Rhine Rivers) and most degraded making robust measures of outcomes hard to achieve. While we have presented data and analyses showing that restoration objectives were met, in general, unequivocal measurements of the success of interventions at these spatial and temporal scales is challenging because of the cost and the inherent variability introduced when collecting a range of long‐term data. This problem continues to elicit tension between scientific considerations and management practicality, and inevitability different definitions of restoration success (Cooke et al. 2017).

Coarse woody habitat restoration is a viable technique to increase population size of large‐bodied fishes in large degraded rivers, assuming there is connection to a source population. Our study delivers on the call for longer‐term, larger‐scale, research projects by several authors over the past two decades (Fausch et al. 2002, Palmer et al. 2005). Indeed, while this study was costly to implement (millions of AUD$), in comparison to the restoration ‐intervention programs that such projects underpin (which can reach tens or hundreds of billions of dollars), the return on the research investment is clearly large. Finally, in an era when delivery of environmental flows is increasingly being used to restore fish populations, restoration that complements flow should have broad appeal and application potential, particularly in areas where flows are limited by human needs, availability of water and/or infrastructural constraints.

## Data Availability

Data are available from the Dryad Digital Repository: https://doi.org/10.5061/dryad.7344f3r
