# Supplementary material for: Increased population size of fish in a lowland river following restoration of structural habitat
Source: Ecol Appl. 2019 Apr 4;29(4):e01882. doi: 10.1002/eap.1882 (PMC6849704; doi:10.1002/eap.1882)
Supplement: Supplementary file 1 [file EAP-29-na-s001.pdf]

Supporting Information. Lyon, J. P., T. J. Bird, J. Kearns, S. Nicol, Z. Tonkin, C. R. Todd, J. O'Mahony, G. Hackett, S. Raymond, J. Lieschke, A. Kitchingman, and C. J. A. Bradshaw. 2019. Increased population size of fish in a lowland river following restoration of structural habitat. *Ecological Applications.*

Appendix S1: Supplementary Material.

Table S1. Summary of data collected to estimate population growth (collected in the intervention and two reference reaches).

N_t	annual electrofishing surveys, mark-recapture data, research angler data
individual age	annual counts of young-of-the-year juveniles, back-calculated age-structure data from otolith collections, stocking data
deaths	mortality from radio tags, capture-mark-recapture data, angler data for fishery-dependent surveys
migration	capture-mark-recapture data (fishery-dependent and -independent), radio tracking movement data

Table S2. Site summary information in order of sampling. We amalgamated *populations 2* and *3* due to the smaller number of samples.

	**Number of sites sampled**						
**Reach description**	**2007**	**2008**	**2009**	**2010**	**2011**	**2012**	**2013**
Population 1	171	225	169	178	170	170	209
Populations 2 and *3*	30	67	49	39	65	28	39
Population 4	134	132	132	128	123	121	122
**TOTAL**	**335**	**424**	**350**	**345**	**358**	**319**	**370**

Table S3. Summaries of target species surgically implanted with radio-tags

Species	Min weight (g)	Max weight (g)	Average weight (g)	Total number
Murray cod	13	43000	3783	689
Golden perch	252	4074	1466	466

Table S4. Summary of target species captured and tagged from 2007 to 2013. Numbers of individuals tagged and recaptured are given in parentheses. *Populations 2* and *3* have been combined due to the smaller samples.

	Population 1		***Populations 2* and *3***		***Population 4***	
	**Murray cod**	**Golden perch**	**Murray cod**	**Golden perch**	**Murray cod**	**Golden perch**
**2007**	**64** (60, 3)	**51** (51, 0)	**21** (19, 0)	**28** (28, 0)	**870** (456, 238)	**237** (187, 44)
**2008**	**176** (124, 6)	**100** (95, 2)	**129** (113, 1)	**123** (122, 1)	**822** (450, 295)	**369** (313, 49)
**2009**	**97** (68, 6)	**32** (30, 1)	**128** (119, 1)	**42** (40, 2)	**675** (690, 312)	**274** (164, 60)
**2010**	**127** (103, 8)	**57** (48, 6)	**187** (107, 1)	**64** (55, 2)	**1028** (258, 201)	**333** (287, 35)
**2011**	**70** (52, 9)	**44** (40, 1)	**256** (216, 4)	**141** (130, 3)	**389** (115, 69)	**258** (238, 24)
**2012**	**116** (97, 8)	**114** (104, 5)	**73** (58, 6)	**61** (60, 1)	**623** (229, 123)	**725** (674, 61)
**2013**	**70** (53, 5)	**95** (94, 1)	**138** (67, 3)	**60** (58, 1)	**1 253** (256, 124)	**546** (498, 57)
**TOTAL**	**720 (616, 45)**	**493, 462, 16)**	**932 (669, 16)**	**519 (493, 10)**	**5 660 (2554, 1364)**	**2742 (2361, 330)**

Table S5. Summary of the model estimates outlining probabilities of transition and 95% confidence intervals. *Lower* and *Upper* refer to the lower and upper 95% Bayesian credible intervals. *Trans* [1,1] refers to the probability of a fish that was in *population 1* in a particular year remaining in *population 1* the following year. *Trans* [1,2] similarly refers to the probability of a fish moving from *population 1* to *population 2*. MC = Murray cod, GP = golden perch.

			95% credible interval	
Transition		Estimate	Lower	Upper
**MC**		**mean**	**2.50%**	**97.50%**
Trans[1,1]	Int->Int	0.968	0.94	0.986
Trans[2,1]	RR1->Int	0.004	0.001	0.015
Trans[3,1]	RR2->Int	0.03	0.022	0.043
Trans[1,2]	Int->RR1	0.002	0	0.011
Trans[2,2]	RR1->RR1	0.965	0.931	0.987
Trans[3,2]	RR2->RR1	0.01	0.004	0.021
Trans[1,3]	Int->RR2	0.011	0.006	0.019
Trans[2,3]	RR1->RR2	0.007	0.003	0.021
Trans[3,3]	RR2->RR2	0.93	0.89	0.957
Trans[1,4]	Int	0.019	0.002	0.048
Trans[2,4]	RR1	0.024	0.002	0.062
Trans[3,4]	RR2	0.03	0.001	0.073
**GP**				
Trans[1,1]	Int->Int	0.93	0.896	0.964
Trans[2,1]	RR1->Int	0.005	0.001	0.014
Trans[3,1]	RR2->Int	0.047	0.031	0.072
Trans[1,2]	Int->RR1	0.006	0.002	0.013
Trans[2,2]	RR1->RR1	0.895	0.851	0.945
Trans[3,2]	RR2->RR1	0.051	0.036	0.07
Trans[1,3]	Int->RR2	0.03	0.02	0.043
Trans[2,3]	RR1->RR2	0.041	0.026	0.063
Trans[3,3]	RR2->RR2	0.859	0.811	0.919
Trans[1,4]	Int	0.034	0.004	0.067
Trans[2,4]	RR1	0.059	0.002	0.109
Trans[3,4]	RR2	0.043	0.001	0.086

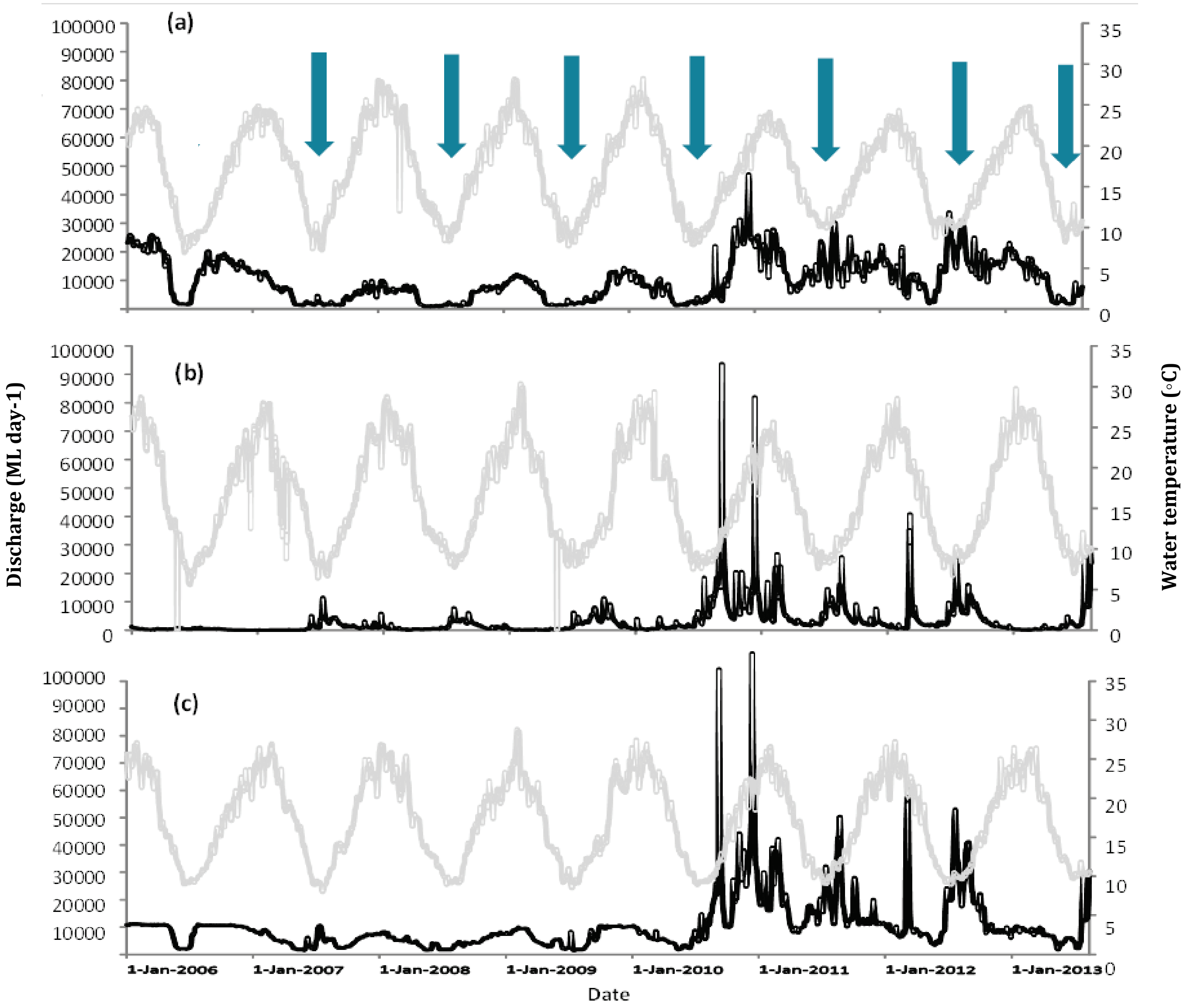

Figure S1. Discharge (ML/day) and temperature (degrees Celsius) within (a) *population 1*, (b) *population 3*), and (c) *population 4*. Grey lines indicate water temperature, black lines indicate flow and arrows indicate sampling. Lake levels for *population 2* are not shown.

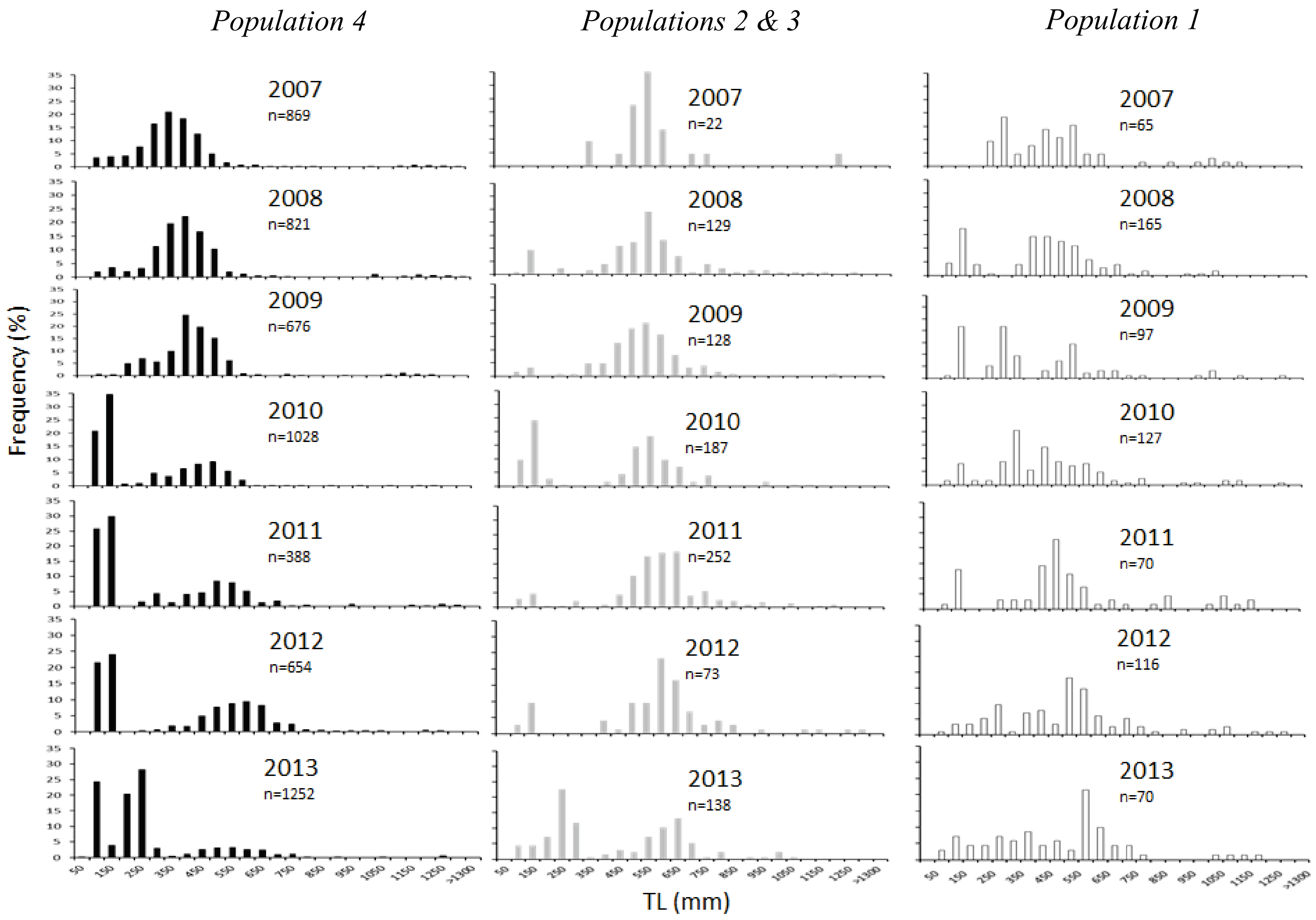

Figure S2. Length-frequency distribution of Murray cod collected during electrofishing surveys of each of the study reaches from 2007 – 2013. Numbers (*n*) of fish collected in each year are also presented. Note: *populations 2* and *3* have been combined due to the low numbers captured.

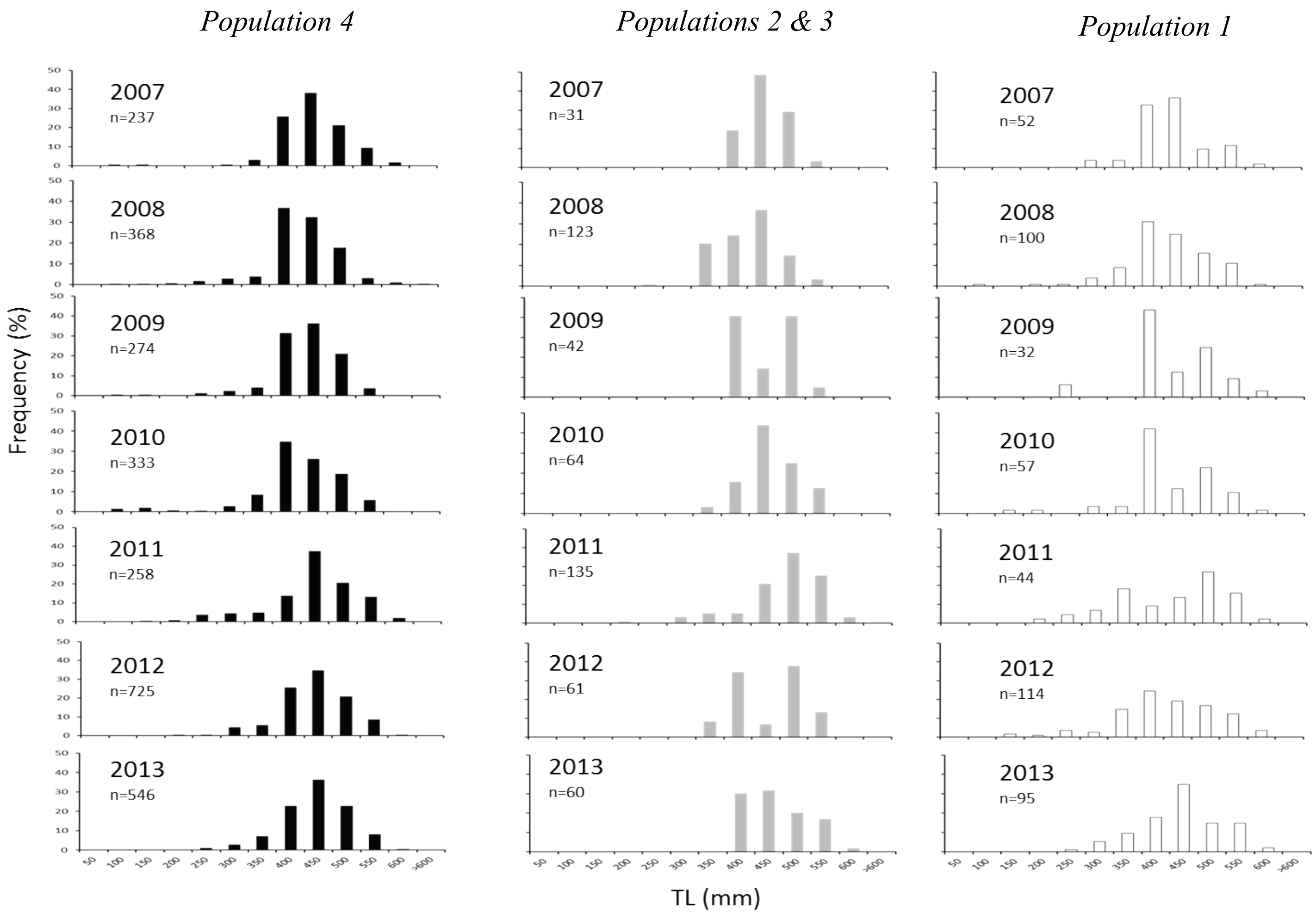

Figure S3. Length-frequency distribution of golden perch collected during electrofishing surveys of each of the study reaches from 2007 – 2013. Numbers (*n*) of fish collected in each year are also presented. Note: *populations 2* and *3* have been combined due to the low numbers captured.

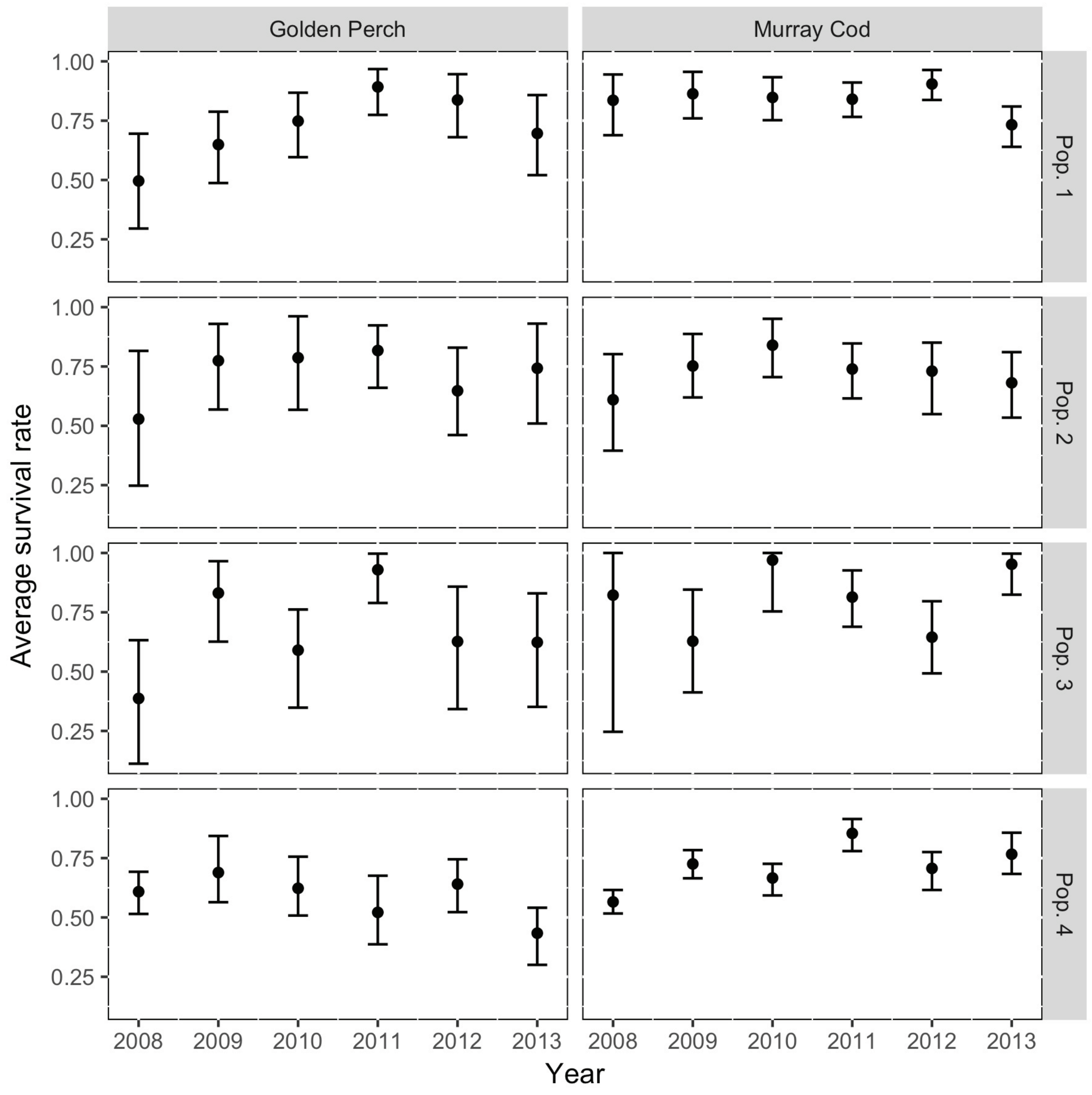

Figure S4. Estimated survival probabilities of Murray cod and golden perch > 200 mm. Error bars represents the 95% credible intervals around the annual estimates. Note: estimates begin in 2008 due to few recaptures in the first year.

Note S1

Monitoring design consultation

We developed our monitoring design through a consultative process with stakeholders and agency decision-makers to ensure it provided information that could be used directly by practitioners. This involved a review of currently known information on interactions between submerged wood and fish, and assessing the limitations of this information for use in management. Through stakeholder workshops, we determined which new knowledge was required, and identified the metrics that would assist decision makers to (1) determine the successes and failures of this restoration type, and (2) provide information that they could use to guide restoration and native fish management in other locations. We concluded that while there is evidence that native fish used restored woody habitats, it was unclear if this use was the result of a simple redistribution of existing individuals or a true increase in population size. We also determined that understanding the processes of survival, recruitment, immigration and emigration that can lead to changes in population size is an essential precursor to answering management questions regarding the importance of nearby source populations, hatchery stocking to supplement recruitment, and fishing mortality.

Note S2

Supplementary Methods

Our modeling approach followed several of the primary assumptions of CMR analyses. First, we assumed no movement to or from the main study reaches, due to the presence of dams and weirs at several of the main entry points. However, we assumed that fish could move with varying levels of ease between the four different reaches. Next, we assumed no tag loss; as the double-tagging employed allowed us to replace single tags that had been lost. Based on previous work, we also assumed that and that tag-induced mortality was low (Bird *et al* 2014) and that tagging did not induce any significant avoidance behavior. We used a state-space framework (King 2012) to separate the observation and process components of our model. Given a set of N fish that are tagged over T evenly-spaced capture-recapture occasions at times $t = 1, \ldots, T$, we denote f_i ($f_i \in 1, \ldots, T$) as the first instance of capture for each individual. We assume that each fish has a set of states of interest that can change over the duration of the study. In general, we use the notation s_i to represent the sequence of states s_{it} ($t = f_i, \ldots, T$) for individual i over the T sampling occasions.

Process model

We initially describe a_{it} as the 'aliveness' state and add more states as we developed the model. We defined the alive/dead state as $a_{it} = 1$ if individual i is alive at time t and $a_{it} = 0$ otherwise. We used the standard Cormack-Jolly-Seber model to describe transitions between alive and dead states, assuming that the states a_{it} are Bernoulli-distributed, exchangeable random variables. The probability of fish i being alive at time t is conditional on the survival parameter μ and whether or not that fish was alive in the previous year, so that

$$\Pr(a_{it} = 1 | a_{it-1}, \mu) = \mu \, a_{it-1}.$$

We additionally allowed μ to vary over time, between different fish ages and between stream sections using a logistic regression.

Adding states to the process model accounts for individual variation in capture probabilities over time. In our case, we have three additional states of interest. First, the study area includes four distinct sections, with varying environments and populations. Second, we are interested in how detection and survival differ across ages. Finally, for the subset of individuals carrying radio tags, we want to estimate the rate at which tags become

undetectable, either through removal from the system or through tag failure. Here we introduce state vectors for each of these factors into our existing model for the observation data.

Stream section: We denote b_{it} as the stream section where individual i was recorded at each t. As with a_{it}, every time an individual is observed during the study, we update b_{it}, which remains unobserved whenever an individual is not detected or captured within a year. To model how live individuals transition between stream sections, we assume that the sequence of states b_{it} are exchangeable random multinomial variables with 4×4 transition matrix η^1, where η^1 has elements

$$\eta^1_{jk} = \Pr(b_{it} = k \mid b_{it-1} = j, a_{it-1} = 1).$$

we use the notation *1* in η^1 to distinguish the matrix of transitions for live individuals versus the matrix of probabilities of dead individuals, η^0 which is all 0's except for the probability of remaining dead. Because we assume that dead individuals do not move, we used the 4×4 identity matrix η^1 when $a_{it} = 0$.

To account for individual changes in age (and therefore, capture probabilities —Bird et al. 2014, Lyon et al. 2014), we modelled changes in individual age over time using sequential observations of how individuals grew in length (L_{it}) to estimate the parameters of a von Bertalanffy growth curve (separately for each reach where data were sufficient). We modelled growth based on the three-parameter von Bertalanffy growth function for asymptotic growth, where the length of individual i at time t is given by:

$$L_{it} = L_\infty - (L_\infty - L_0)(1 - e^{-k_{si}}),$$

where L_∞ is the asymptotic size, L_0 is the size at which individuals enter the population (defined as 0 here), and k is the unitless growth parameter. Based on this growth equation, we modelled the observed change in length between two capture occasions using the von Bertalanffy growth curve:

$$g_{i(t_2 - t_1)} \sim Normal((L_\infty - L_{ic-1})(1 - e^{-k_{si}}), \sigma^2),$$

where $g_{i(t_2 - t_1)}$ is the expected growth of individual i between times t_2 and t_1, and k_{si} is the growth rate for state (population) s_i. Based on these parameters, we could back-calculate the expected age at first capture for each fish, and therefore, the influence of this age on the probability of detection. Finally, we included a state for whether each fish carried an active telemetry tag, which we modelled as a random Bernoulli variable:

$$D_{it+1} \sim Bernoulli(\gamma D_{it}),$$

where D_{it} records whether individual i carried an active telemetry tag at time t, and γ is the probability of that tag remaining active.

Data models

We treated all capture data as independent, exchangeable Bernoulli variables with probability of success conditional on a capture parameter p that can itself depend on the states s_{it}. We additionally assumed that observations are conditional both on fish having a recognizable tag and being within the study area for sampling in each of the T observation periods. Because the Murray River has many side branches, we assumed that fish can migrate out of the sampling area, thus rendering them unavailable for sampling.

However, we assumed that this movement is random and therefore confounded with p.

Electrofishing

We electrofished within discrete sampling areas and did not cover the entire fishable area. However, because fish migrate (Koehn and Harrington 2006), we assumed that movement in and out of sampling areas was random. Let e_i represent the sequence of electrofishing observations for individual i over sampling occasions $t = f_i, \ldots, T$, with $e_{it} = 1$ if a fish was observed at time t and 0 if it was not. The probability of a fish being captured at time t is:

$$\Pr(e_{it} = 1 \mid e_{it\text{-}1}, \mu_t) = p^e{}_t \, a_{it}$$

where $p^e{}_t$ denotes the probability of capture by electrofishing for live fish in year t.

Anglers

We use the notation r_i to denote the vector of angler resightings for individual i. As with electrofishing, we assumed that a fish must be alive to be captured, so

$$\Pr(r_i = 1 \mid a_{it\text{-}1}, \mu_t) = p^r{}_t \, a_{it}.$$

However, unlike the electrofishing data, we assumed that anglers were distributed throughout the sampling area. As well, anglers provided data on the fates of their catches. Thus, we were able to update $a_{it} = 0$ for fish that were killed on capture or released.

Telemetry

The vectors x_i and y_i provide the series of observations by telemetry survey and logger-tower records for each fish. We denote the time of implantation for each telemetry-tagged fish as F_i, and z_i as the tag-activity state for each fish, with $z_{it} = 1$ if a tag is implanted and active and 0 otherwise. We initially set the activity state for tags to be 0 for all times beyond their guaranteed failure dates, and ignored any detections following this date. For both telemetry and logger-tower observations, we assumed that the detections are exchangeable Bernoulli random variables with probability of detection $p^{telem}{}_t$ and $p^{log}{}_t$, respectively.

However, for telemetry observations, we assumed that detections were not conditional on whether or not the fish was alive. This is based on the observation that much of the river is shallower than the maximum depth of 3 m at which radio telemetry is thought to attenuate in fresh water, and presupposes that live and dead fish have similar detection rates. We also ignored temporary and permanent movement of radio-tagged fish. Because the telemetry tags emit a unique signal for fish that have remained motionless for > 1 week, we were also able to update the aliveness history a_i whenever a tag was detected, whether the fish was alive or dead.

Logger towers

By contrast, we assumed that logger towers could only detect fish if they were alive, because fish needed to swim within the vicinity of a radio tower to be recorded. As a consequence, $p^{log}{}_{it}$ is less of a detection probability than a probability that a radio-tagged fish will swim past a tower and be detected. In our initial models, we made no distinction between detections by any of the towers in the study area. However, we did use the sequence of transitions between towers for each fish to infer which stream section a fish was in during the electrofishing sampling period.

Incorporating latent states in the observation models

We use an individually-based logistic regression to model the dependence of capture probabilities on stream section, age and tag status. We here define the fully parameterised models which include all combinations of latent state data, which were then simplified by removing various combinations of latent states. Given the large number of possible parameter combinations, we take a pragmatic approach to modelling combinations of parameters and

note that when we removed parameters from one capture model we also removed it from the others. For electrofishing and angler recaptures, we include terms for the p in each stream section and year. Next, we assumed a linear relationship between age and capture probabilities that is consistent between all 4 streamsections. This gives

$$\text{logit}(p^{e}_{it}) = \beta^{e}_{0} + \beta^{e}_{y} + \beta^{e}_{b} + \beta^{e_{\text{age}}}\ \text{age}_{it}$$

where β^{e}_{0} is the intercept parameter, β^{e}_{y} is vector of year-specific parameters, β^{e}_{b} is a length 4 vector of stream section-specific parameters, and$\beta^{e_{\text{age}}}$ is a coefficient describing the linear relationship between capture and age. We defined a similar model for p^{e}. For telemetry and logger observations, we kept the dependence on stream section and year, removed the age parameter, and included a parameter for whether or not the fish was alive. We also conditioned telemetry detections on whether or not the tag borne by a fish was active, such that

$$p^{telem}{}_{it} = \begin{cases} \beta^{t}_{0} + \beta^{t}_{b_{it}} + \beta^{t}_{a_{it}} + \beta^{t}_{y} & \text{if } z_{it} = 1 \\ 0 & \text{otherwise} \end{cases}$$

Inference and model evaluation

We used Markov chain Monte Carlo to sample from the posterior distribution of each model. We gave all parameters in the logistic regression model diffuse Normal (0, 0.0001) priors, and gave dirichlet priors to the rows of the transition matrix η^{1}. For each model, we ran three chains of 120,000 samples in parallel, disregarding the first 80,000 chains and keeping every 40th sample for the remaining 40,000 chains in each, for a total of 3,000 samples from each posterior distribution. Instead, we used Bayesian p values and root mean-squared error, both of which provide measures of how distant model-predicted values are from observed data (goodness-of-fit). We calculated both of these goodness-of-fit indices for each of the data sources when present in a model, and report how these values vary among models.

Because we accounted for major sources of variation in capture probabilities (i.e., movement, variation in size, and mortality), we estimated population size N_y for each year of the study by modelling the observed number of captures n_y within each age class as a binomial random variable with probability of capture defined by p_y and a vague Gamma (0.01, 0.01) prior distribution for the true population size N_y. We used Markov chain Monte Carlo sampling software JAGS in the R package R2jags.

References

Bird, T., J. Lyon, S. Nicol, M. McCarthy, and R. Barker. 2014. Estimating population size in the presence of temporary migration using a joint analysis of telemetry and capture–recapture data. Methods in Ecology and Evolution 5:615-625.

King, R. 2012. A review of Bayesian state-space modelling of capture–recapture–recovery data. Interface Focus.

Koehn, J. D., and D. Harrington. 2006. Environmental conditions and timing for the spawning of Murray cod (*Maccullochella peelii peelii*) and the endangered trout cod (*M. macquariensis*) in southeastern Australian rivers. River Research and Applications 22:327-342.

Lyon, J. P., T. Bird, S. Nicol, J. Kearns, J. O'Mahony, C. R. Todd, I. G. Cowx, and C. J. Bradshaw. 2014. Efficiency of electrofishing in turbid lowland rivers: implications for measuring temporal change in fish populations. Canadian Journal of Fisheries and Aquatic Sciences 71:878-886.

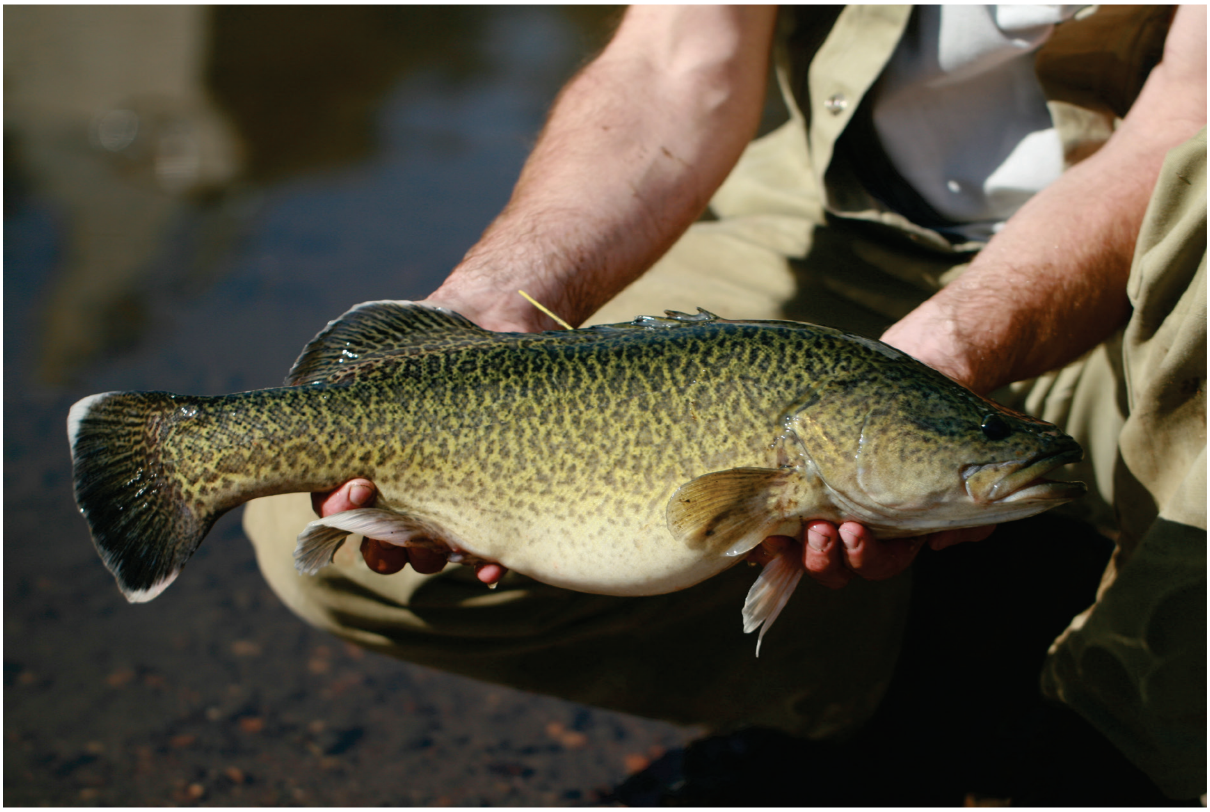

Plate S1: A tagged Murray cod (*Maccullochella peelii)*. Photo Credit: J. Lyon.

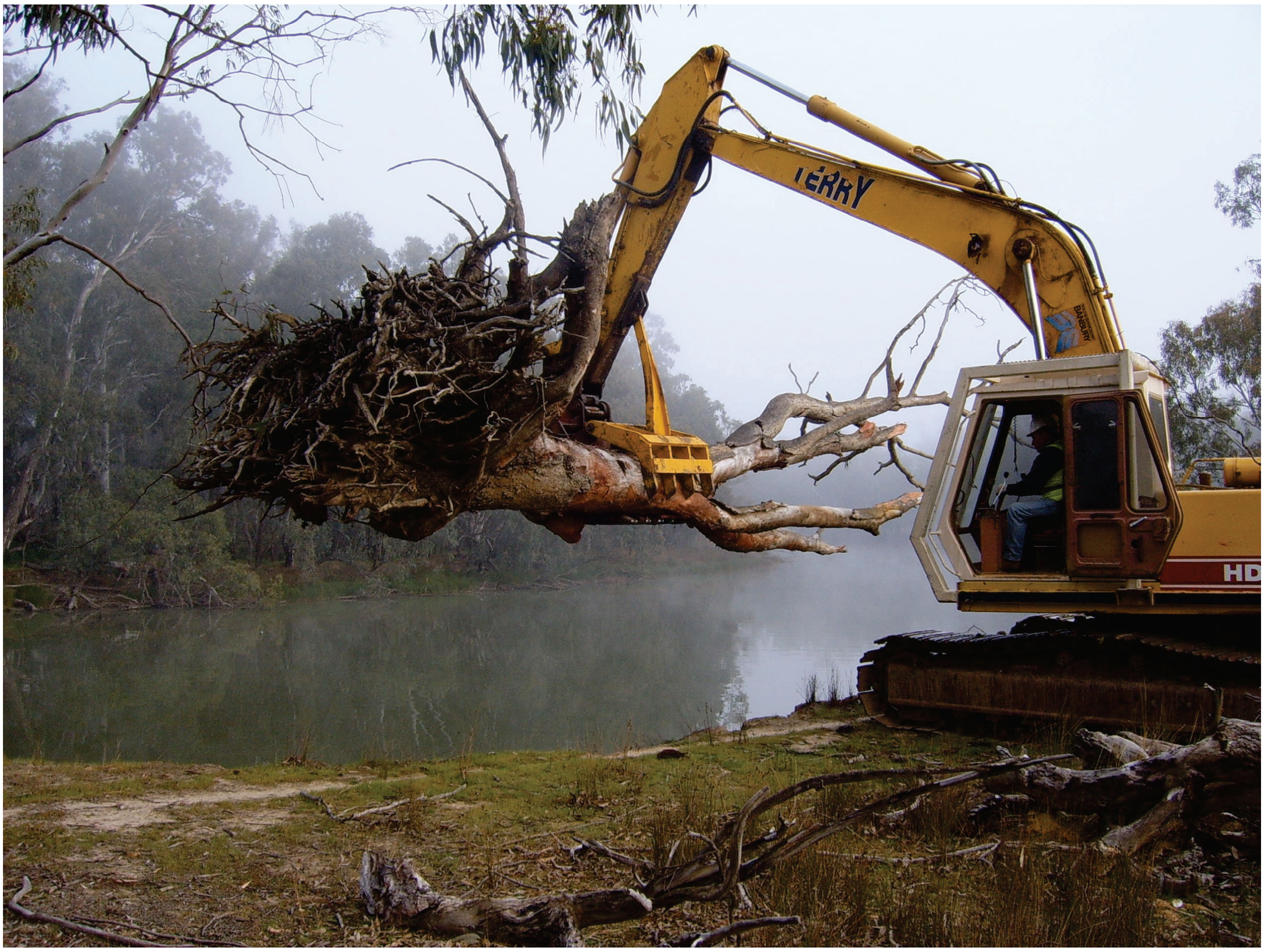

Plate S2: Habitat restoration being done in the Intervention Reach. Photo Credit: M. Casey.

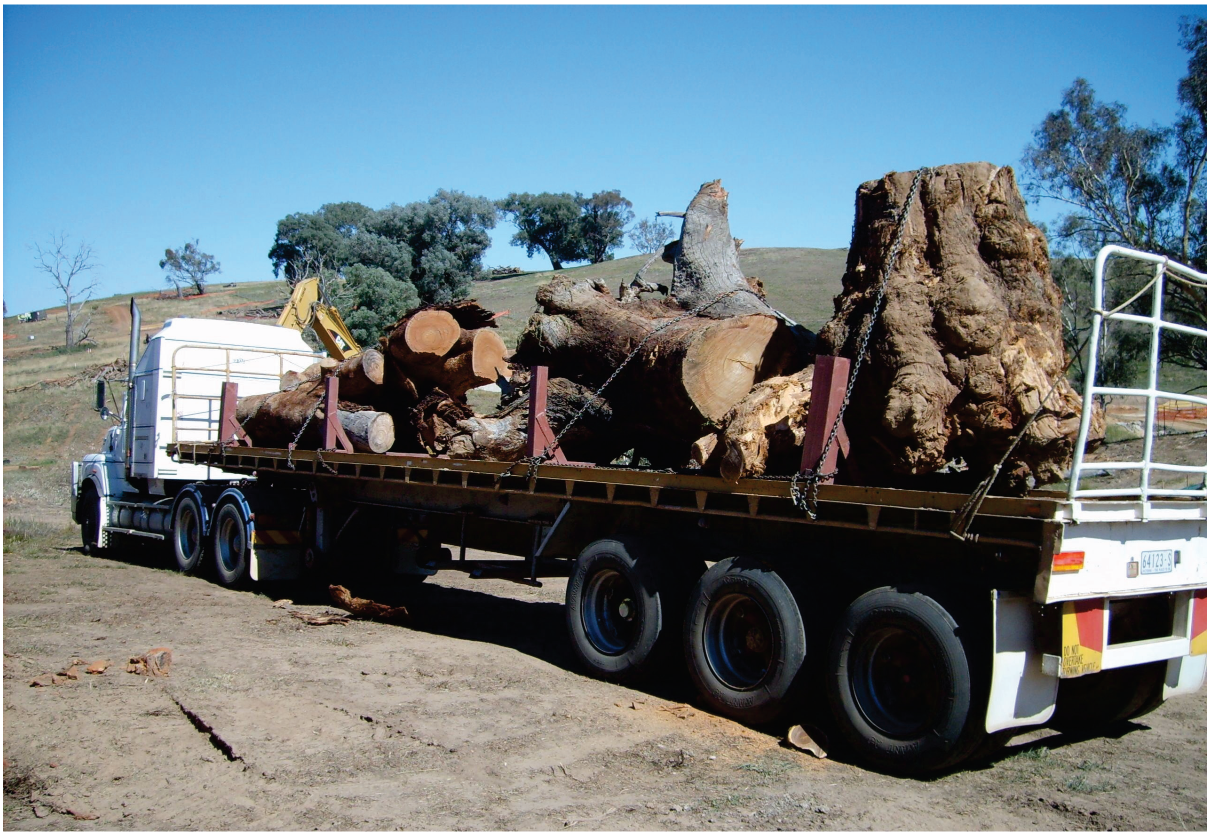

Plate S3: Coarse woody habitat being transported to the Intervention Reach. Photo Credit: M. Casey

.
